# Serping1 associated with α-synuclein increase in colonic smooth muscles of MPTP-induced Parkinson’s disease mice

**DOI:** 10.1038/s41598-024-51770-9

**Published:** 2024-01-11

**Authors:** Min Hyung Seo, Soo-Hwan Kim, Sujung Yeo

**Affiliations:** 1 Department of Meridian and Acupoint, College of Korean Medicine, Sang Ji University, Wonju, 26339 Republic of Korea; 2 Division of Biological Science and Technology, Yonsei University, Yonseidae 1 Gil, Wonju, 26493 Republic of Korea; 3 Research Institute of Korean Medicine, College of Korean Medicine, Sangji University, Wonju, 26339 Republic of Korea

**Keywords:** Molecular biology, Neuroscience

## Abstract

Patients with Parkinson’s disease (PD) have gastrointestinal motility disorders, which are common non-motor symptoms. However, the reasons for these motility disorders remain unclear. Increased alpha-synuclein (α-syn) is considered an important factor in peristalsis dysfunction in colonic smooth muscles in patients with PD. In this study, the morphological changes and association between serping1 and α-syn were investigated in the colon of the 1-methyl 4-phenyl 1,2,3,6-tetrahydropyridine-induced chronic PD model. Increased serping1 and α-syn were noted in the colon of the PD model, and decreased serping1 also induced a decrease in α-syn in C2C12 cells. Serping1 is a major regulator of physiological processes in the kallikrein-kinin system, controlling processes including inflammation and vasodilation. The kinin system also comprises bradykinin and bradykinin receptor 1. The factors related to the kallikrein-kinin system, bradykinin, and bradykinin receptor 1 were regulated by serping1 in C2C12 cells. The expression levels of bradykinin and bradykinin receptor 1, modulated by serping1 also increased in the colon of the PD model. These results suggest that the regulation of increased serping1 could alleviate Lewy-type α-synucleinopathy, a characteristic of PD. Furthermore, this study could have a positive effect on the early stages of PD progression because of the perception that α-syn in colonic tissues is present prior to the development of PD motor symptoms.

## Introduction

Patients with Parkinson’s disease (PD) have gastrointestinal motility disorders as common non-motor symptoms, including dysphagia, defecatory dysfunction due to impaired colonic transit, and constipation^1–5^. Although it has been reported that lesions of the autonomic nervous system are responsible^1^ and Lewy pathology affects the gastrointestinal tract, causing enteric inflammation in the initiation and/or progression of PD^6^, the pathological cause of non-motor symptoms is still unclear. However, with the recent discovery of α-synuclein (α-syn) deposition in the gastrointestinal tract, an increasing number of studies have shed light on the strong connection between pervasive α-syn aggregations and PD^6–9^.

The enteric nervous system (ENS) can serve as a duct for propagation of α-syn, initiating the characteristic spread of degeneration throughout the central nervous system^7,10^. The α-syn depositions are generated in the mucosal nerve fibers and submucosal and myenteric plexuses throughout the ENS^11^. The prevalence of Lewy-type α-synucleinopathy staining may reliably distinguish PD colon from control (CTL) using suitable immunohistochemical (IHC) staining methods^12,13^. α-Syn pathology in colonic tissues has also been observed prior to the development of characteristic PD motor symptoms^8^.

The serine proteinase inhibitor family G1 (serping1), also called C1 inhibitor (C1INH), is a major controller of the kallikrein–kinin system and controls complement C1 and plasminogen activation, thereby contributing to inflammation^14^. Loss of function mutation of *serping1* results in hereditary angioedema, leading to fatal intermittent acute swelling in the gastrointestinal tract and larynx^15^. However, *serping1* is upregulated in patients with active tuberculosis^16^, monocytes from HIV-1 + patients, and monocytes following treatment with IFN-α, IFN-β, and IFN-γ^17^. Following traumatic brain injury, an inflammatory response in the central nervous system activates astrocytes and microglia, resulting in gliosis, and increased *serping1* expression, a characteristic of pro-inflammation, is observed in astrocytes treated with IL-1α^18^. It has been suggested to have potential as a therapeutic target for neurodegenerative disorders, and it was reported in a previous study that *serping1* is a promising therapeutic target associated with α-syn in PD^19^. Furthermore, it has been reported that there is a strong relationship between *serping1* expression and schizophrenia in cortical thickness^20^.

In the kinin system, bradykinin activates intracellular pathways that increase endothelial junctions, and bradykinin receptor 1 (B1R) may contribute to angioedema attacks upon inflammation^21^. Upon bradykinin stimulation, B1R activates endothelial nitric oxide synthase and leads to local edema and fluid extravasation^22,23^. Neuroinflammatory disorders such as Alzheimer's disease and PD are characterized by chronic inflammation and loss of vascular integrity^24^. Stimulation of B1R increases the secretion of pro-inflammatory cytokines, IL-6, IL-8, intracellular adhesion molecule-1, vascular cell adhesion molecule-1, and monocyte chemoattractant protein-1^25–28^, but decreases the expression of vascular endothelial growth factor required for maintaining vasculature^24^. B1R stimulation also induced the loss of occludin expression at tight junctions and increased vascular permeability, as well as breakdown of the blood–brain barrier (BBB)^24^. It has been reported that bradykinin levels increase significantly in brain injury^29^ and the release of bradykinin is associated with the loss of BBB integrity^30–33^. Activation of B1R is associated with neuroinflammatory processes in vitro and in vivo^34–36^ and also with multiple sclerosis^37^ and Alzheimer's disease^38^.

In this study, based on the report that the MPTP (1-methyl 4-phenyl 1,2,3,6-tetrahydropyridine) is a representative dopamine neuron toxin in the ENS inducing gastrointestinal (GI) dysfunction^4^, the association between serping1, a major controller of the kinin system, and α-syn in PD colon was investigated in an MPTP-induced chronic PD model. This study hypothesized that non-motor PD symptoms such as decreased phasic rectal contraction, weak abdominal strain, and paradoxical sphincter contraction on defecation in constipation^5^ can be considered to be smooth muscle contraction-relaxation defects macroscopically, and serping1 could also be associated with gastrointestinal motility disorders of PD, as colon consists of a smooth muscle-like vascular system. In light of the changes in serping1, alterations in α-syn and kinin system-related factors, bradykinin and B1R, were also studied in C2C12 cells in this study.

## Results

### MPTP-induced PD model in brain

**Figure 1:**
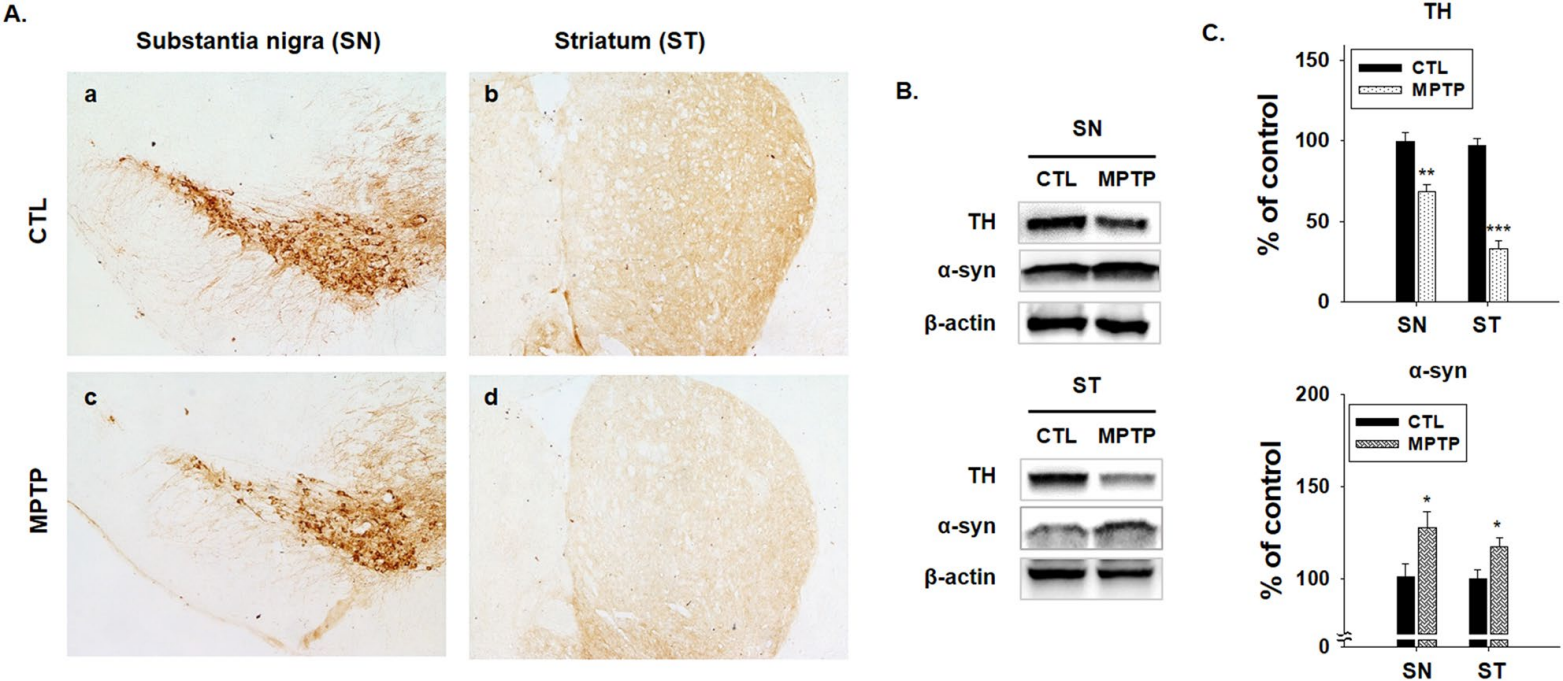
Reduction of tyrosine hydroxylase (TH) in substantia nigra (SN) and striatum (ST) in the 1-methyl-4-phenyl-1,2,3,6-tetrahydropyridine (MPTP)-induced Parkinson’s disease model. (**A)** Immunohistochemical staining of TH (a-d) was performed in SN and ST regions in control (CTL) and MPTP groups (n = 3/group). TH decreased in SN and ST regions in the MPTP group (c, d). (**B)** Immunoblotting of TH in SN and ST and of α-syn in SN. TH decreased in SN and ST in the MPTP group. α-Syn increased in SN in the MPTP group. (**C)** Immunoblotting results presented as a bar graph. (n = 3, *p < 0.05, **p < 0.005, ***p < 0.001) .

PD presents with characteristics of dopaminergic neuron degeneration in the substantia nigra (SN) and TH deficiency in the striatum (ST)^39^. In this study, the chronic PD model induced by MPTP for 4 weeks showed significantly decreased TH expression levels in the SN and ST (Fig. 1, *p* < 0.005). However, α-syn increased remarkably in the SN and ST regions in immunoblot analysis (*p* < 0.05); these results were quantified in a histogram (Fig. 1B,C). This result shows the characteristics of PD.

### The morphological changes in colon in the MPTP-induced PD model

**Figure 2:**
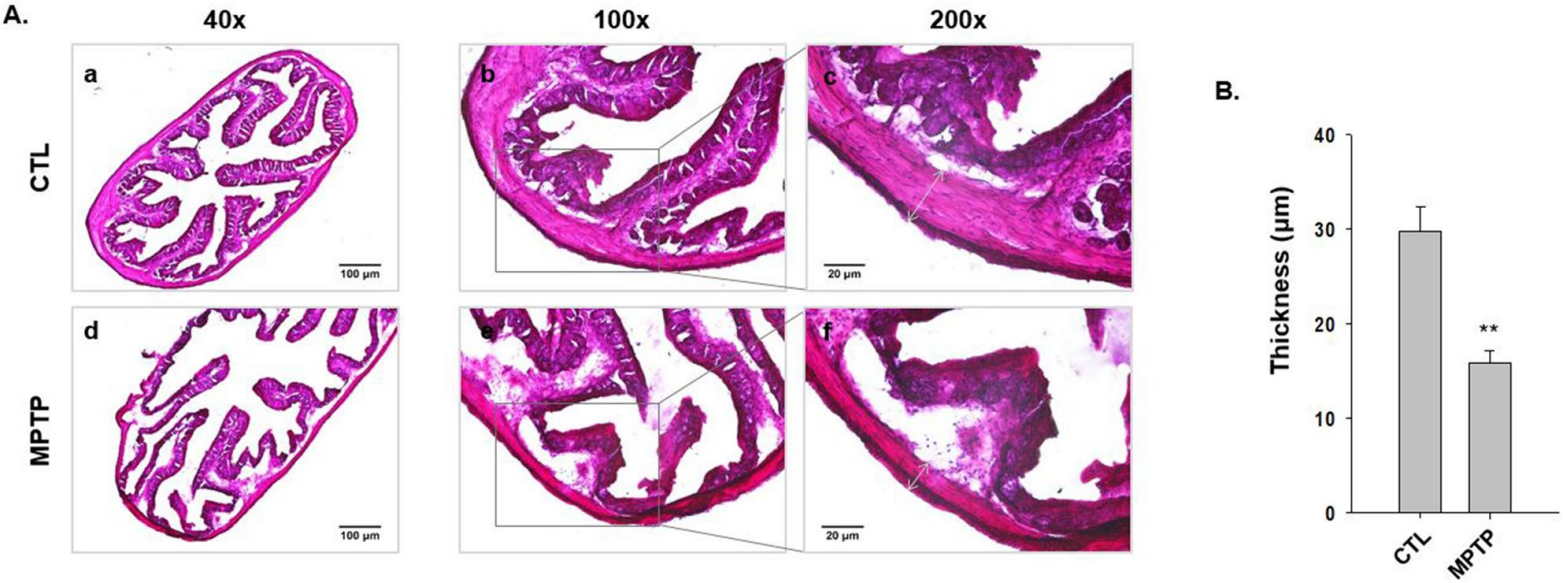
The morphological change of thickness of colonic smooth muscle in 1-methyl-4-phenyl-1,2,3,6-tetrahydropyridine (MPTP)-induced Parkinson’s disease. (**A**) Hematoxylin and eosin staining in control (CTL) and MPTP groups (n = 3/group). The colonic matrix of the CTL group was sturdier than the matrix of the MPTP group (a, d) and the thickness (c, f; indicated by bidirectional arrows) of colonic smooth muscle decreased significantly in the MPTP group. (**B**) Thickness of colonic smooth muscle in the MPTP group versus control (n = 3, **p < 0.005).

To investigate the morphological changes in the colon in the MPTP-induced PD mouse model, H&E staining was performed using colon tissues from the CTL and MPTP groups (n = 3/group). The staining results are shown in Fig. 2A. The tissue matrix in the MPTP group was less sturdy than that in the CTL group. The thickness of the surrounding GI tract tissue, composed of smooth muscle that causes contraction relaxation surrounding the colon tissue, was randomly selected and measured (Fig. 2A; indicated by bidirectional arrows). The results were plotted in a graph indicating that the thickness of the surrounding colon tissue in the MPTP group decreased significantly (Fig. 2B, p < 0.005).

These morphological changes can affect the motility function of the colon, and it can be deduced that contraction-relaxation could be strongly associated with serping1 related to vascular endothelial contraction-relaxation. Thus, serping1 in the colon tissue was investigated.

### Serping1 and α-syn in colon smooth muscle of the MPTP-induced PD model

**Figure 3:**
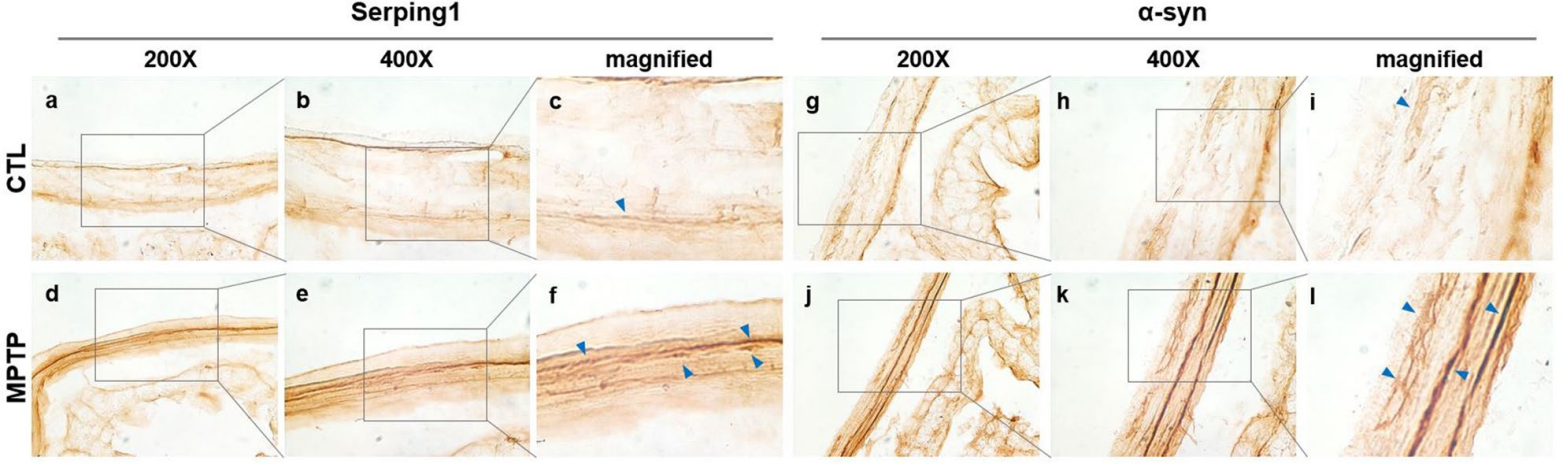
Increased serping1 and α-synuclein (α-syn) in colonic smooth muscle in the Parkinson’s disease model. Immunohistochemical analysis of serping1 (**a**-**f**) and α-syn (**g**-**l**) conducted in colon tissue in control (CTL) and 1-methyl-4-phenyl-1,2,3,6-tetrahydropyridine (MPTP) groups (n = 3/group). Serping1 increased in the MPTP group (c, f); increased serping1 indicated by arrows. α-Syn increased in the MPTP group (i, l; α-syn indicated by arrows).

Immunohistochemical analysis of serping1 in the colon was performed in the CTL and MPTP groups (n = 3/group). Based on the finding that α-syn deposits are discovered in the GI tract, α-syn in the colon was also analyzed immunohistochemically, and the expression patterns of serping1 and α-syn were compared in the CTL and MPTP groups (Fig. 3). Serping1 and α-syn expression patterns were similar in the surrounding colon tissue (Fig. 3d and j), and the expression levels of serping1 and α-syn appeared to be increased in the MPTP group (Fig. 3a,d; g and j).

**Figure 4:**
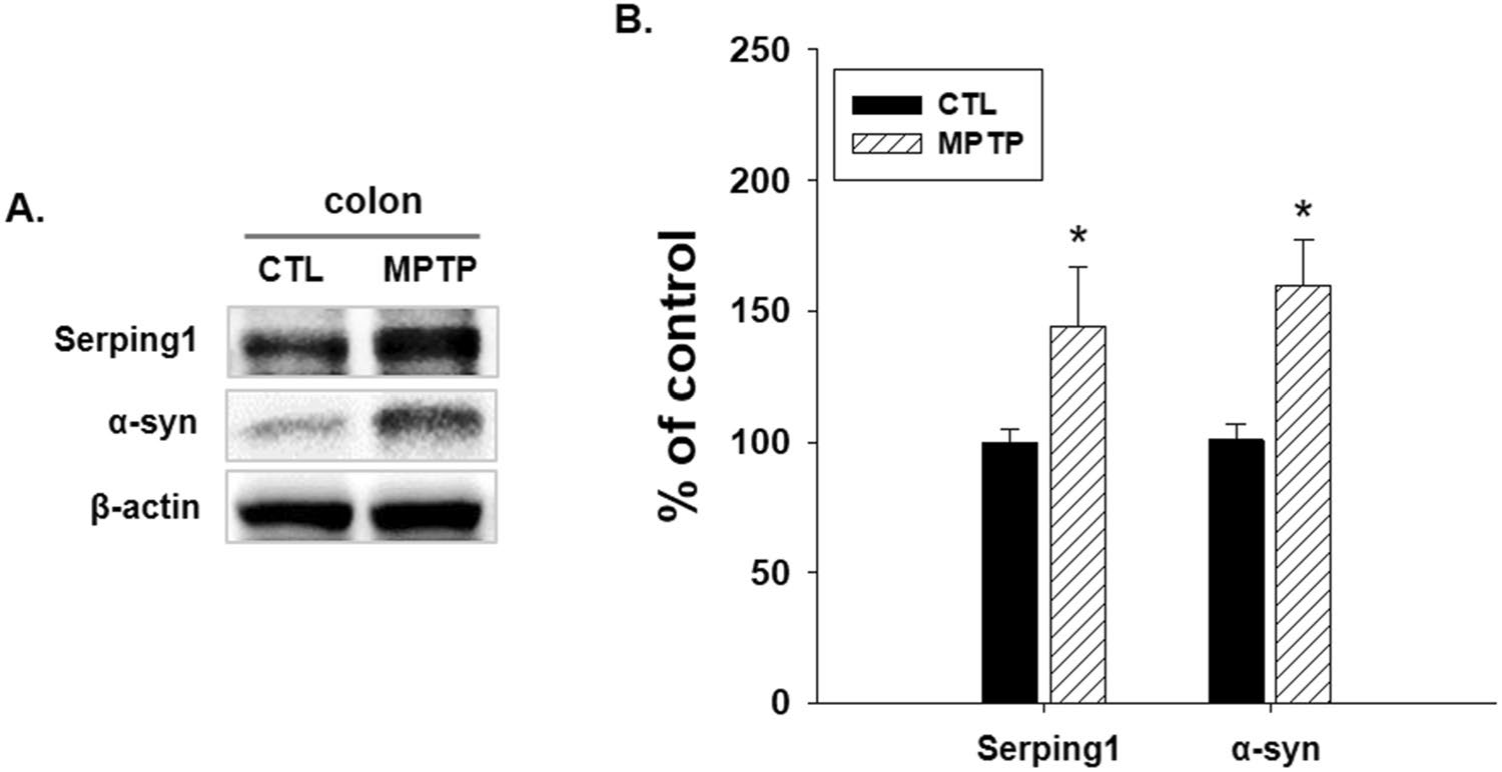
Increased serping1 and α-synuclein (α-syn) in colon in the Parkinson’s disease model. (**A**) Western blot analyses of serping1 and α-syn were investigated in colon in control (CTL) and 1-methyl-4-phenyl-1,2,3,6-tetrahydropyridine (MPTP) groups. Serping1 and α-syn increased in the MPTP group. (**B**) Bar graph of the immunoblot analysis results, showing significantly increased serping1 and α-syn (n = 3, *p < 0.05).

The expression levels of serping1 and α-syn were also analyzed by immunoblotting in the CTL and MPTP groups, the results of which were presented in a bar graph (Fig. 4). Serping1 and α-syn expression levels were significantly increased in the MPTP group (Fig. 4A, B, p < 0.05).

**Figure 5:**
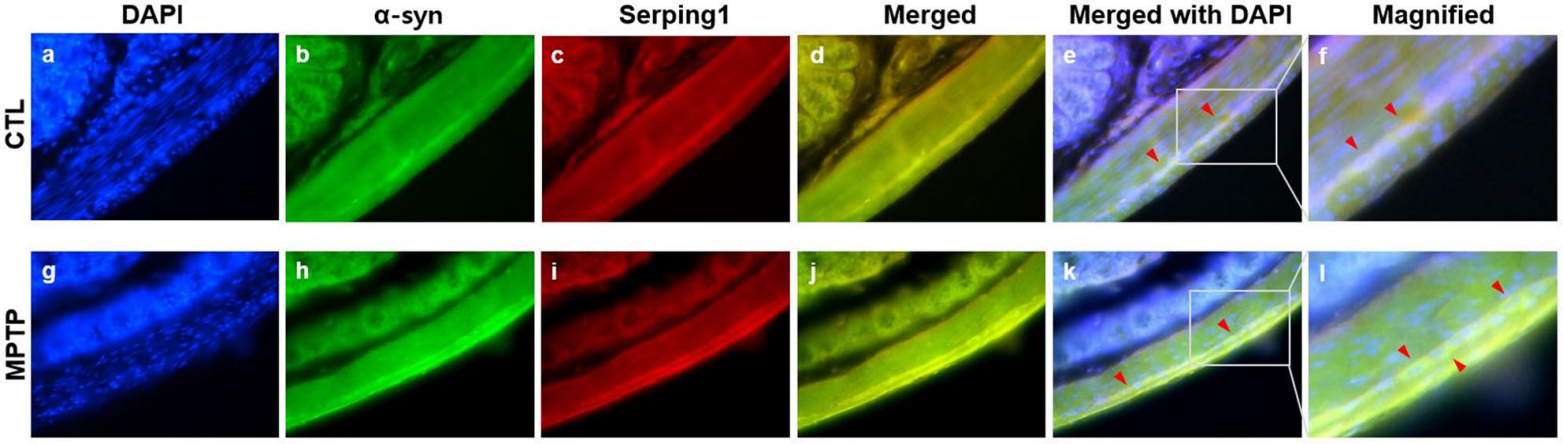
Immunofluorescence microscopy analysis of serping1 and α-synuclein (α-syn) in colonic smooth muscle in the Parkinson’s disease model. Immunofluorescence analysis of serping1 and α-syn in control (CTL; **a**-**f**) and 1-methyl-4-phenyl-1,2,3,6-tetrahydropyridine (MPTP; **g**-**l**) groups (n = 3/group). Nuclei were stained with DAPI (**a**, **g**; 400X). α-Syn staining is shown in panels **b** and **h**, and serping1 staining is shown in panels **c** and **i** in CTL and MPTP groups. Merge of α-syn and serping1 staining (**d**, **j**; 400X) and merge with DAPI (**e**, **k**; merged serping1 and α-syn indicated with arrows). Panels **f** and **l** and magnifications of panels **e** and **k**, respectively. More clear and brighter merged serping1 and α-syn staining surrounding nuclei in the MPTP group than in the CTL group (f, l; merged serping1 and α-syn indicated with arrows).

Because of the similarity of expression patterns of serping1 and α-syn (Fig. 3), immunofluorescence microscopy analyses of serping1 and α-syn were also performed in the CTL and MPTP groups (n = 3/group) to identify the correlation (Fig. 5). The colocation of serping1 and α-syn was confirmed in the merged panels (Fig. 5d, j), and more brightly co-located serping1 and α-syn in the MPTP surrounding the nuclei were also examined (Fig. 5f,l).

These results confirmed the correlation between serping1 and α-syn in the colon in the PD model, and it can be deduced that serping1 can affect GI motility dysfunction with α-syn. Therefore, it was necessary to investigate the changes in the factors related to serping1 in the colon of the MPTP group.

### Changes in factors related to serping1 in colon of the MPTP-induced PD model

**Figure 6:**
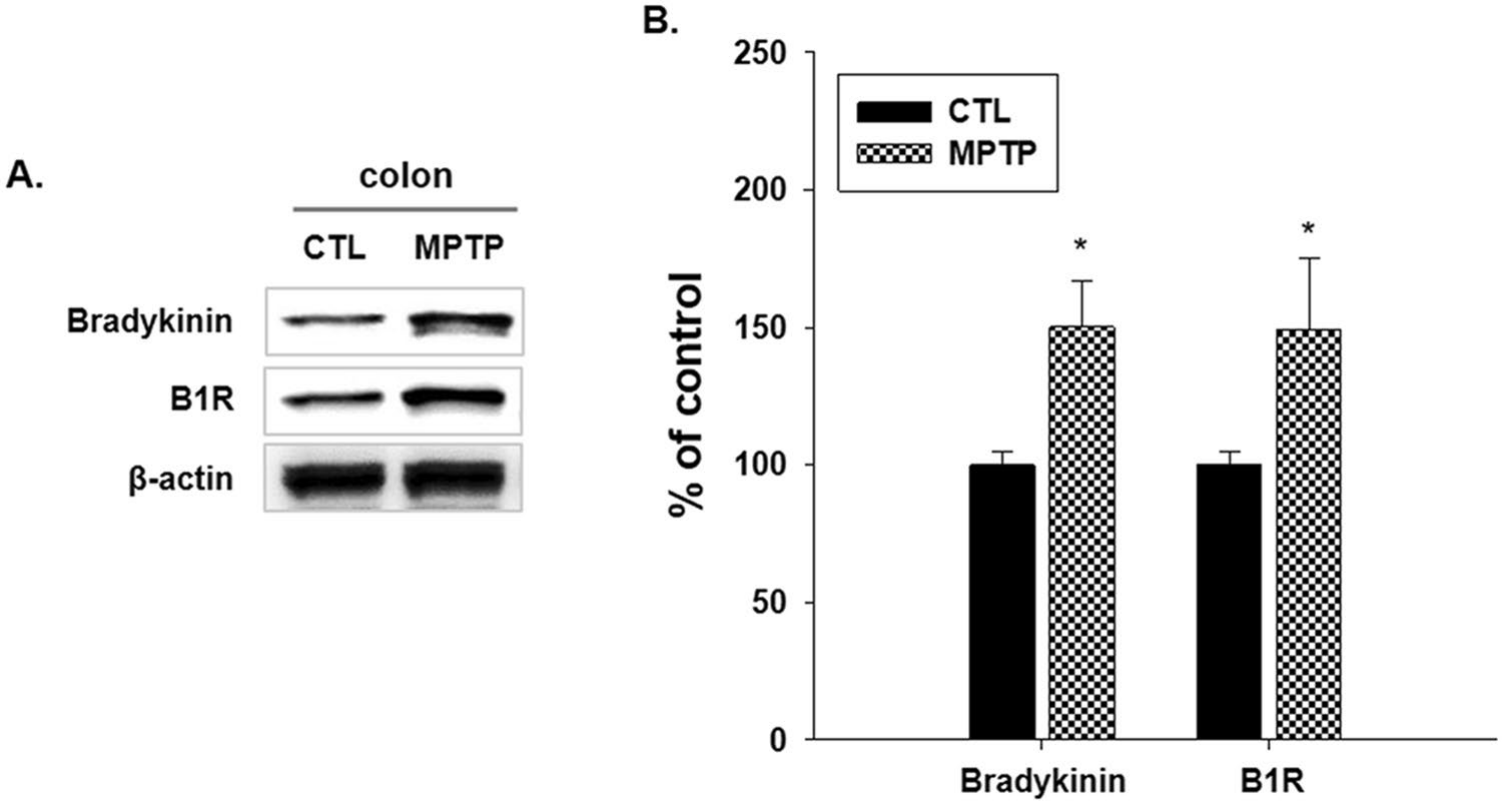
Western blot analysis of changes in factors related to serping1 in colon in 1-methyl-4-phenyl-1,2,3,6-tetrahydropyridine (MPTP)-induced Parkinson’s disease. (**A**) The factors related to the kinin system regulated by serping1, bradykinin and bradykinin B1 receptor (B1R), were investigated. B1R increased significantly in the MPTP group. (**B**) Bar graph of the Western blotting analysis results (n = 3, *p < 0.05).

The factors associated with serping1 in the kinin system, bradykinin and B1R, were also examined in the colons of the CTL and MPTP groups (n = 3/group). Immunoblotting analysis indicated that bradykinin and B1R expression levels were significantly increased in the MPTP group (Fig. 6). The immunoblot results (Fig. 6A) are displayed as a bar graph, showing significantly increased bradykinin and B1R levels (Fig. 6B).

Increased expression levels of bradykinin and B1R, related to serping1 were confirmed in the MPTP-induced PD model. It was also necessary to investigate the changes in bradykinin and B1R caused by serping1 as well as the correlation between serping1 and α-syn.

### Changes in factors related to serping1 and α-syn in C2C12 cells

**Figure 7:**
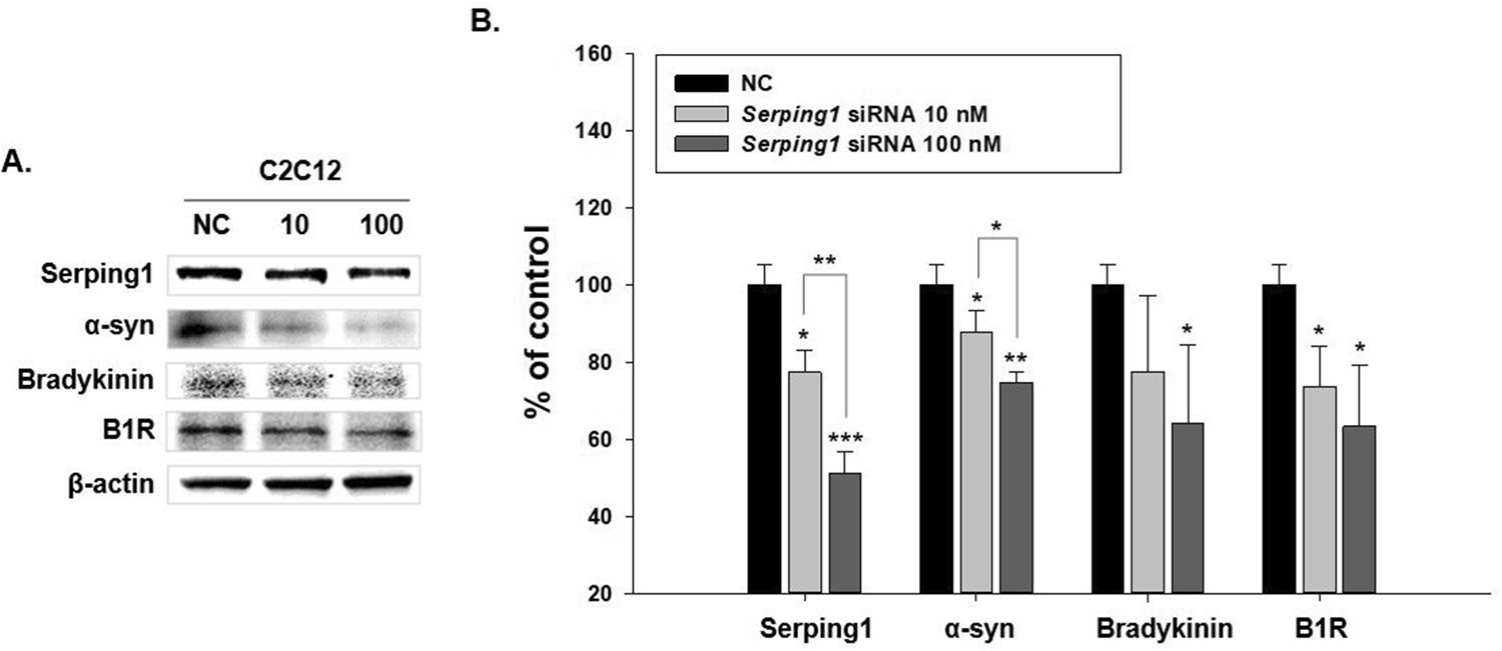
Changes in factors related to the kinin system and α-synuclein (α-syn) according to the extent of *serping1* knockdown in C2C12 cells. (**A**) Immunoblot analysis of down-regulation of *serping1* expression by *serping1* siRNA and the levels of α-syn and the factors associated with the kinin system, bradykinin and bradykinin B1 receptor (B1R). The negative control (NC) group was transfected using non-targeting siRNA. We applied 10 and 100 nM of the siRNA targeting *serping1*. The NC group was also treated with 100 nM of siRNA. (**B**) Bar graph of the immunoblotting results, showing that knockdown of *serping1* expression correlated with reduction of the level of α-syn, bradykinin and B1R, significantly in C2C12 cells. (n = 3, *p < 0.05, **p < 0.005, ***p < 0.0005).

To examine the changes in bradykinin, B1R, and α-syn caused by serping1, immunoblotting analysis was performed in C2C12 cells with *serping1* knockdown. The negative control (NC) duplexes of short interfering RNA (siRNA) were utilized to compare the siRNA against *serping1* because the NC duplexes represent a non-targeting siRNA. We confirmed that *serping1* expressions decreased in C2C12 cells transfected using *serping1* siRNA (10,100 nM) compared to the expression levels associated with NC siRNA (Fig. 7). Decreasing serping1 levels corresponded with significant decreases in the expression levels of α-syn, bradykinin, and B1R; the results were illustrated in a histogram (Fig. 7).

The results indicate that serping1 affected the α-syn and the kinin system factors, bradykinin and B1R. Therefore, it can be deduced that serping1 is an important factor affecting α-syn increase in the PD colon and affects the kinin system related to contraction-relaxation inducing GI mobility dysfunction.

## Discussion

In this study, serping1 and α-syn expression levels were increased in the colon of the MPTP-induced chronic PD model. As reports have highlighted a strong connection of pervasive α-syn aggregations in the gastrointestinal tract with PD^6–9^, α-syn was detected in the colon, and the serping1 expression pattern was similar to that of α-syn. The relationship between serping1 and α-syn was also confirmed in vitro, indicating that decreasing serping1 could decrease α-syn and alleviate Lewy-type α-synucleinopathy, which is a characteristic of PD. Lewy bodies and Lewy neurites have been identified in Auerbach’s and Meissner’s plexuses in patients with non-symptomatic sporadic PD^40,41^. Interestingly, the vasoactive intestinal polypeptide neuron system is also primarily involved in the PD process in the gut^42^. The resulting loss of function mutation of *serping1* leads to hereditary angioedema^15^. Furthermore, the early Lewy pathology during the course of PD affects the ENS, even before the pathology manifests in the SN^13,43^. Therefore, it is also worth noting that modulating serping1 in the early stages of PD could have a positive effect on PD progression, as α-syn in colonic tissues was discovered prior to the development of PD motor symptoms^8^.

An increase in serping1 expression, which is a characteristic of pro-inflammation, has been reported in astrocytes in post-traumatic brain injury^18^. It could be deduced that GI motility dysfunction is caused by α-syn depositions in nerve fibers in the ENS and by the inflammation increasing serping1. However, this study also revealed that increasing serping1 can increase α-syn in colonic smooth muscle moved by peristalsis, which is deeply related to GI motility dysfunction, in that reducing *serping1* decreased α-syn in C2C12 cells. Therefore, decreasing serping1 could alleviate GI motility dysfunction, including constipation, and activation of bowel-releasing waste products may slow the progression of PD.

Increased bradykinin and B1R in the kinin system modulated by serping1 enlarge endothelial junctions, and B1R activates endothelial nitric oxide synthase to increase vascular permeability^21–23^. A less sturdy colon tissue matrix, based on these characteristics, in the MPTP-induced PD model (Fig. 2) could be induced by the increased bradykinin and B1R regulated by the increase in serping1. This could increase the propagation of α-syn, initiating the characteristic spread of degeneration^7,10^.

As it has been reported that activation of B1R is associated with neuroinflammatory processes^34–36^ related to neurodiseases^37,38^ and increased secretion of factors related to pro-inflammatory cytokines^25–27^, it can be deduced that B1R modulated by serping1 may be involved in the inflammatory reactions in Alzheimer’s disease, and PD can be considered to be a neuroinflammatory disorder^24^. However, the mechanistic associations between inflammatory factors and B1R were not addressed in this study. It is, therefore, necessary to research the associations precisely in a future study, and it is of considerable relevance that research on the factors related to serping1 can be applied to PD as well as Alzheimer’s disease.

Furthermore, a limitation of this research was its implementation of a murine model, which is not linearly associated with humans. Also, our examination of serping1 and related factors focused only on the GI smooth muscle motility dysfunction associated with PD. However, our results imply the possibility of providing a positive effect in the early stages of PD progression. However, serping1 and related factors in the kallikrein-kinin system require further research in the blood vessels of the GI tract and the ENS, including Auerbach’s and Meissner’s plexuses. The association between the vasoactive intestinal polypeptide neuron system and serping1 is also of interest for further research.

In conclusion, serping1 and α-syn expression levels were increased in the colon of the MPTP-induced chronic PD model. The expression patterns of serping1 and α-syn were similar. It could also be deduced that increasing serping1 induced an increase in α-syn in colonic smooth muscle moved by peristalsis, which is closely related to GI motility dysfunction, including constipation, as reducing serping1 induced decreased α-syn in C2C12 cells. Increased bradykinin and B1R in the kinin system modulated by serping1 enhance endothelial permeability and induce the propagation of α-syn, initiating the characteristic spread of degeneration. The kinin system factors bradykinin and B1R, regulated by serping1 could be also closely associated with pro-inflammatory reactions in Alzheimer’s disease and PD as neuroinflammatory disorders. However, further research is required in this regard.

## Methods

### MPTP mouse model

Male, 4-week-old, C57BL/6 mice (19–21 g; DBL, Korea) were divided into two groups (n = 6/group): control (CTL) and MPTP-treated (MPTP). In the control group, mice were injected with 100 μL of phosphate-buffered saline (PBS) once daily for 4 weeks. In contrast, mice in the MPTP group received intraperitoneal MPTP-HCL injections (20 mg/kg of free base; Sigma-Aldrich, USA) in PBS (100 μL) every 24 h for 4 weeks to produce a chronic model of PD. The mice were anesthetized using Alfaxan® and then perfused with cold PBS through the circulatory system for western blotting. All animal experiments conducted for this study were approved by the Sang Ji University Animal Experimentation Committee (protocol #2021–9). All methods were performed in accordance with the relevant guideline and regulations. The animal study is reported in accordance with ARRIVE guidelines.

### Immunohistochemistry

The brains and colons of each group were perfused with 4% paraformaldehyde and fixed for one day at 4 °C. After fixation, the brains were dehydrated in 30% sucrose buffer for two days at 4 °C. Coronal sectioned brains (40 μm) and sagittally sectioned colons (40 μm) were cut using a cryomicrotome. Immunohistochemical analysis was performed using an ABC kit and a Mouse on Mouse (M.O.M) immunodetection kit (Vector Laboratories, CA, USA). Sections including the ST (striatum) and SN (substan-tia nigra) regions were incubated in H_2_O_2_ with PBS (pH 7.4) and then incubated in blocking buffer. A mouse anti-TH (tyrosine hydroxylase) antibody (1:2000; cat. no. sc-25269, Santa Cruz Biotechnology, USA) was used. Rabbit anti-serping1 (1:2000; cat. no. PAA235Mu01, Cloud-Clone Corp., USA) and rabbit anti-a-syn (1:500; cat. no. NBP2-15,365, Novus Biologicals, USA) antibodies were used for the colon tissues. Thereafter, the sections were treated with biotinylated anti-mouse IgG and an avidin–biotin-peroxidase complex, which reacted with diaminobenzidine hydrogen peroxide. The sections were analyzed using a Nikon X-cite® series 120Q microscope (Nikon, Japan).

### Western blotting

Brain and colon tissues were homogenized in a radioimmunoprecipitation assay buffer (cat. no. IBS-BR009, iNtRON Biotechnology, Inc., Korea) composed of 1% triton X-100 and 0.1% sodium dodecyl sulfate, using a sonicator (Qsonica Q55, USA) on ice for 20 min. C2C12 cells were incubated and homogenized in Tris-Triton cell lysis buffer (GenDEPOT, USA) for 20 min on ice. After centrifugation, tissues and the C2C12 cells at 4 °C for 15 min were separated using 12% sodium dodecyl sulfate–polyacrylamide gel electrophoresis and then transferred to polyvinylidene difluoride membranes (Pall Life Science, USA). The membranes were blocked with 3% BSA, incubated with primary antibodies, and washed with 0.1% Tris-buffered saline containing Tween 20. The membranes were then incubated with a secondary antibody for one hour and washed with 0.1% Tris-buffered saline containing Tween 20.

Mouse anti-TH (1:2000; cat. no. sc-25269, Santa Cruz Biotechnology), rabbit anti-α-syn (1:500; cat. no. NBP2-15,365, Novus Biologicals), rabbit anti-serping1 (1:2000; cat. no. PAA235Mu01, Cloud-Clone Corp.), mouse anti-bradykinin (1:500; cat. no. sc-293304, Santa Cruz Biotechnology), mouse anti-B1R (1:500; cat. no. sc-293196, Santa Cruz Biotechnology), and mouse anti-β-actin (1:5000; cat. no. sc-47778, Santa Cruz Biotechnology) were used as primary antibodies. The ratios of the investigated protein to beta actin on western blotting were presented in histograms.

### Hematoxylin and eosin staining

The fixed colon tissues were sagittally cryo-sectioned (40 μm) and stained using a hematoxylin and eosin stain kit (Vector Laboratories).

### Immunofluorescence microscopy

Sagittal colon tissue cryosections were used for immunofluorescence microscopy analysis of the control and MPTP groups. The sections were fixed in 4% paraformaldehyde and incubated in blocking buffer (5% goat serum in PBS) for 1 h. The samples from both groups were incubated with mouse anti-α-syn (1:500; cat. no. 610787, BD Biosciences, USA) and rabbit anti-serping1 (1:2000; cat. no. PAA235Mu01, Cloud-Clone Corp.) primary antibodies. Goat anti-mouse IgG (H + L) fluorescein isothiocyanate-conjugated (cat. no. CSB-PA980279, Cusabio Technology LLC, USA) and goat anti-rabbit IgG (H + L) tetramethylrhodamine-conjugated (cat. no. A16115, Novex, USA) were used as the secondary antibodies. Finally, cell nuclei were labeled with DAPI (1 μg/mL). The exposure parameters were the same for the control and MPTP groups and the samples were observed using a Nikon X-cite® series 120Q microscope (Nikon, Japan).

### Cell lines and cultures

The C2C12 myoblast cell line was kindly provided by Prof. Sung-Hee Hwang (Department of Pharmaceutical Engineering, Sangji University, Wonju, South Korea) and was cultured under standard culture conditions (5% CO2, 37 °C). Dulbecco’s modified Eagle’s medium (GenDEPOT, USA) containing 10% fetal bovine serum (GenDEPOT, USA) and 100 U/mL of penicillin–streptomycin (Gibco, USA).

### Short interfering RNA knockdown

C2C12 cells were incubated in Opti-MEM™ medium (Gibco, USA) at least one day before siRNA (short interfering RNA) transfection. When the density of the C2C12 cells was 30%, the transfection reagent and *serping1* siRNA were applied (3.5:1). Transfection was performed for 24 h. siRNA against *serping1* (5′-CAC CUA UGU GAA UGC AUC U-3′) and negative control (NC) duplexes (5’-UUC UCC GAA CGU GUC ACG UTT-3’) were used (Bioneer Inc., Korea). The NC duplexes siRNA was utilized as a control to compare the siRNA transfected against *serping1* because the NC duplexes present a non-targeting siRNA.

### Imaging software

ImageJ software (version 1.52a) developed at the National Institutes of Health and the Laboratory of Optical and Computational Instrumentation (University of Wisconsin) was used to analyze the images.

### Statistical analysis

Statistical analyses were performed using Student’s *t*-test and analysis of variance in SPSS 25 software (SPSS Inc. Released 2017, PASW Statistics for Windows, Version 25.0, USA). All values are expressed as means ± the standard error.

### Supplementary Information

Supplementary Information.

## Data Availability

All necessary data are included in the paper.
