# Supplementary material for: Serping1 associated with α-synuclein increase in colonic smooth muscles of MPTP-induced Parkinson’s disease mice
Source: Sci Rep. 2024 Jan 11;14:1140. doi: 10.1038/s41598-024-51770-9 (PMC10784473; doi:10.1038/s41598-024-51770-9)
Supplement: Supplementary file 1 — Supplementary Information. [file 41598_2024_51770_MOESM1_ESM.pdf]

Figure 1B. SN

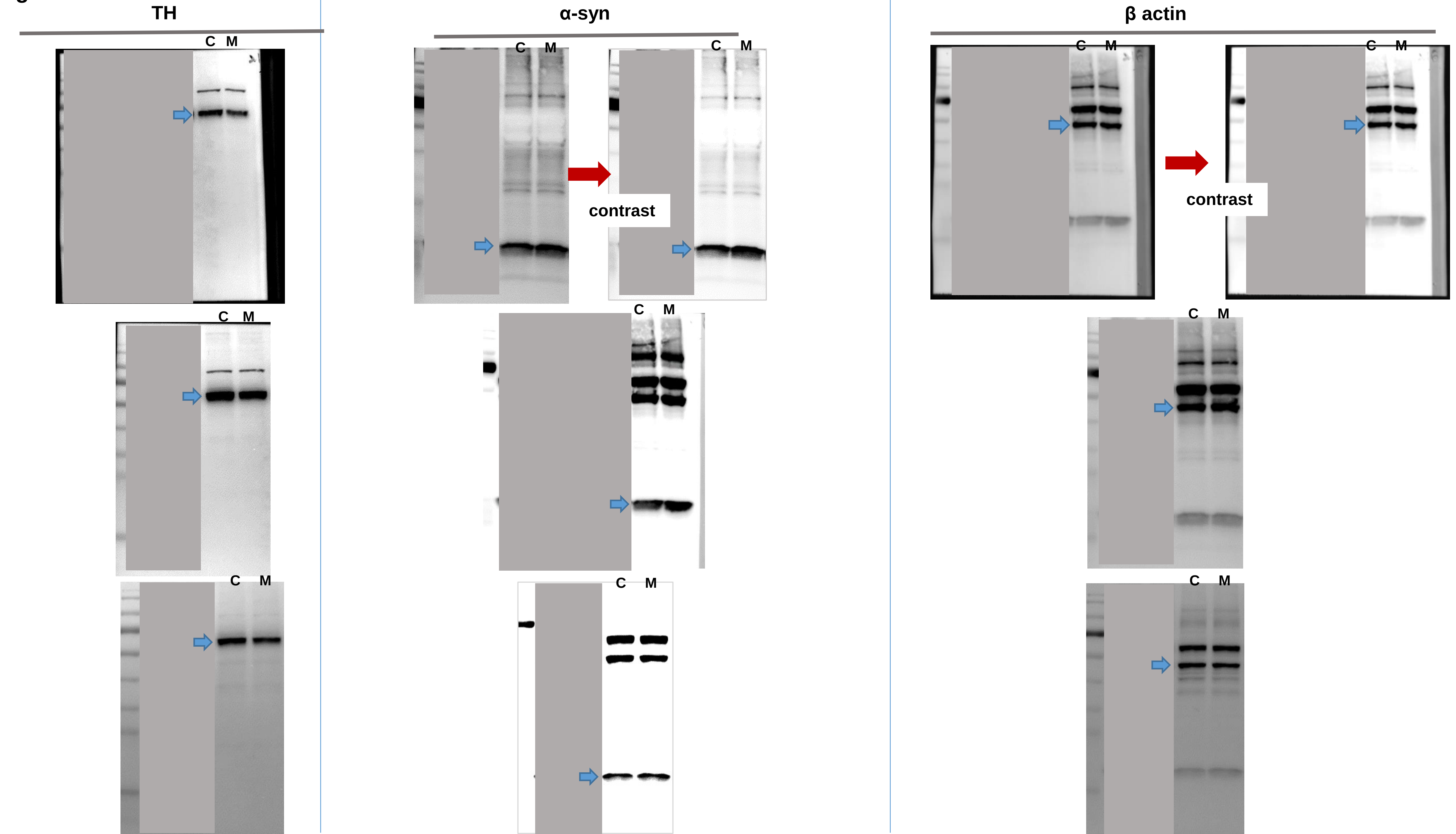

Figure 1B. ST

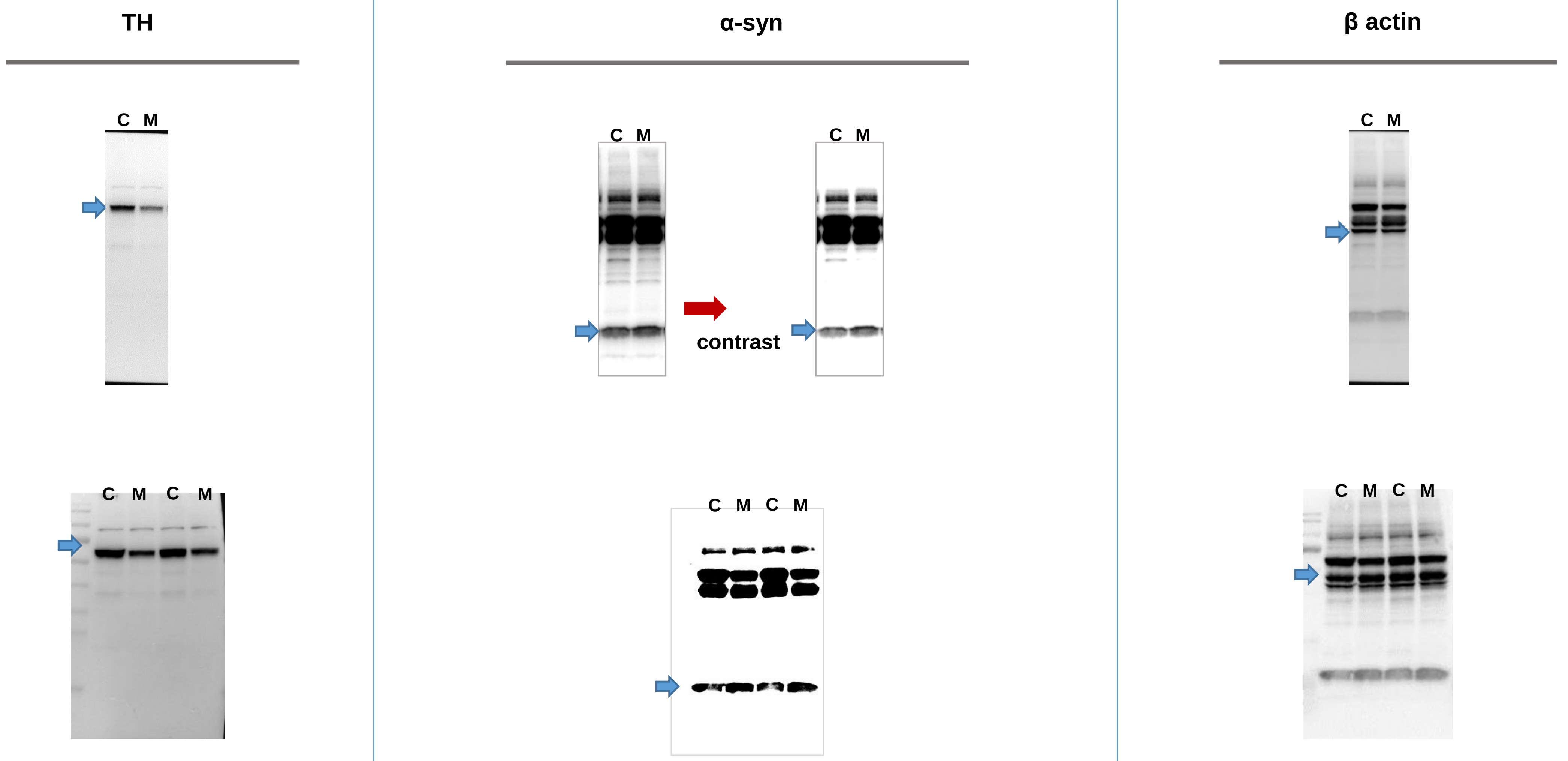

Figure 4

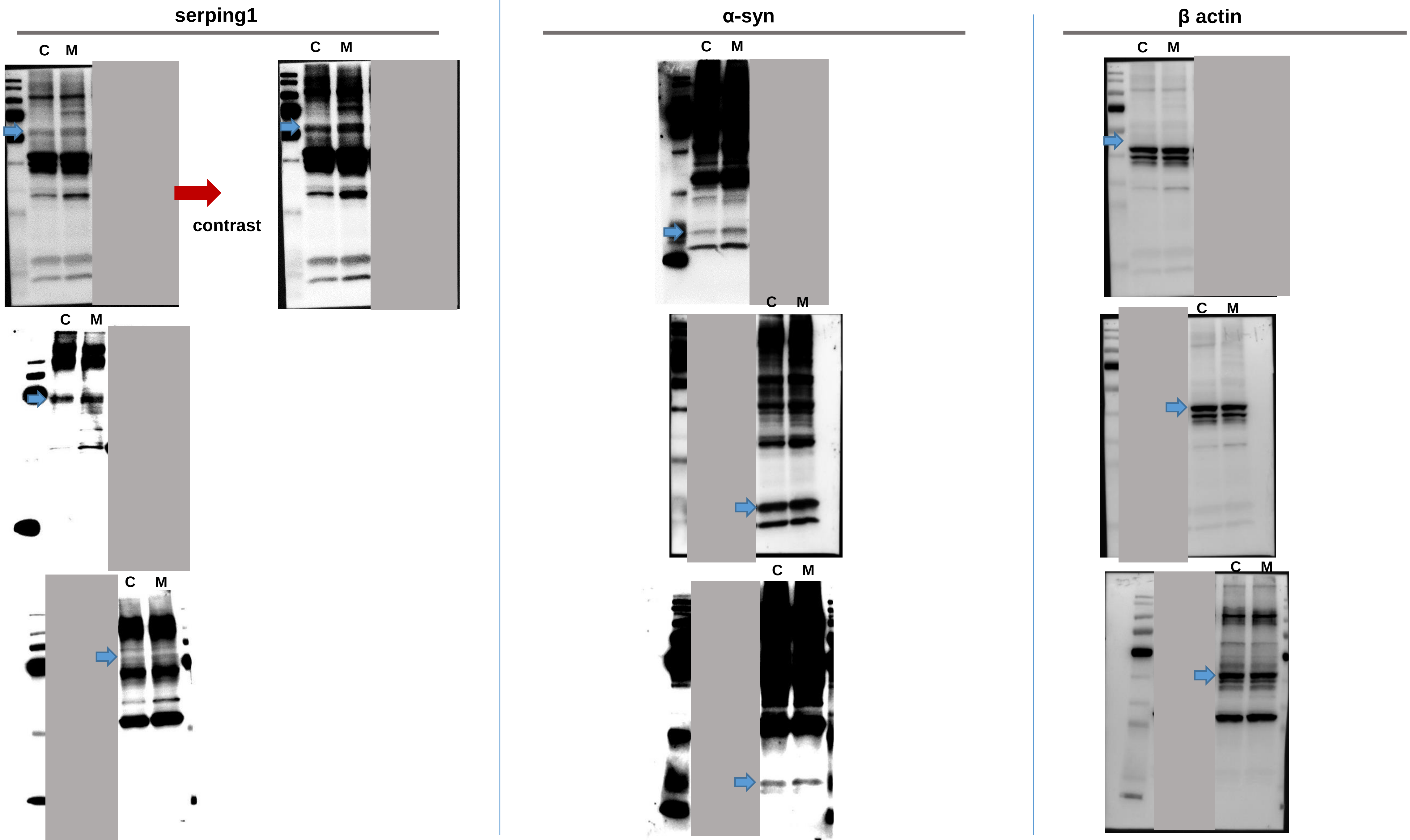

Figure 6

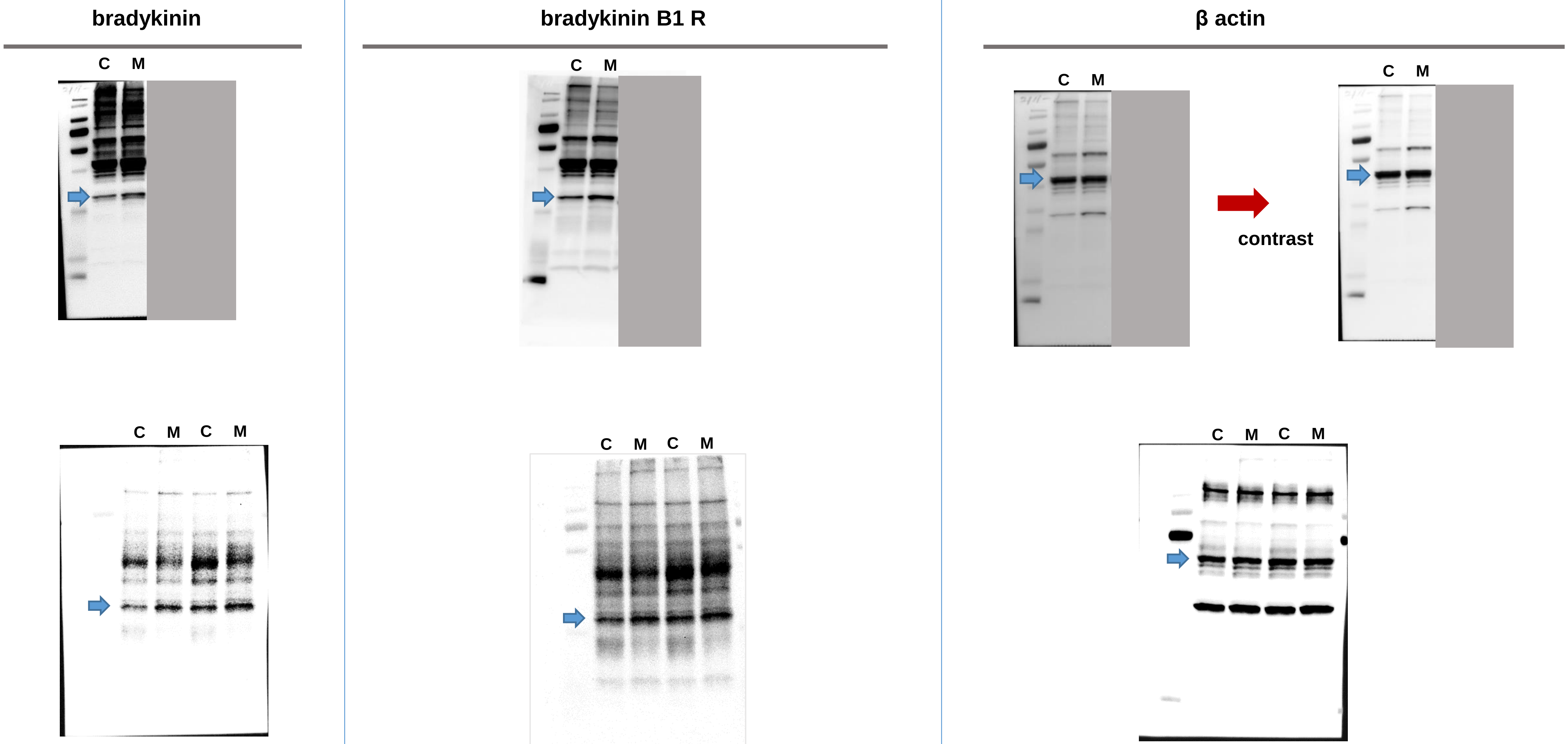
bradykinin
bradykinin B1 R
β actin
C M
C M
C M
C M
contrast
C M C M
C M C M
C M C M

Figure 7

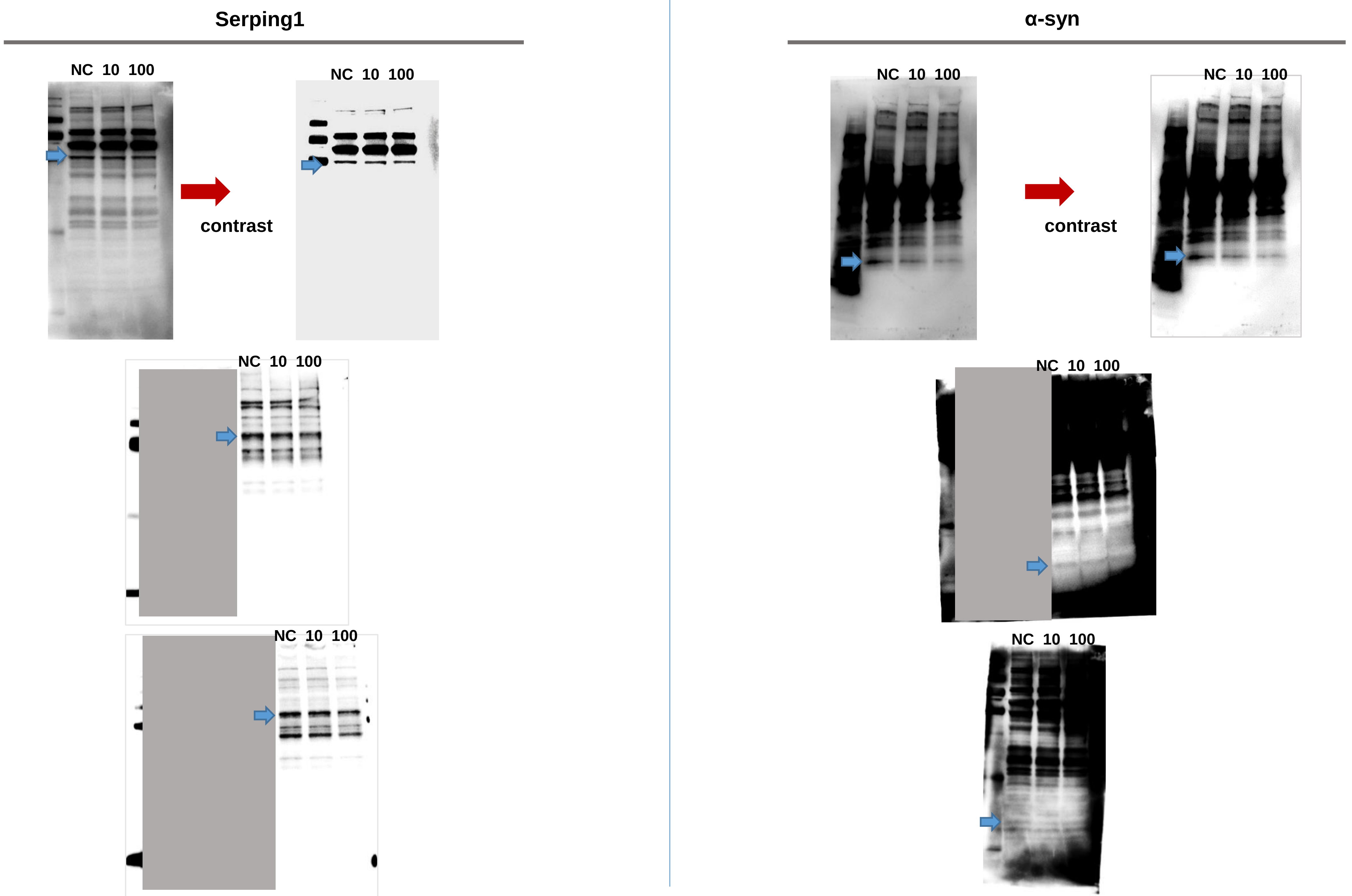
Serping1
NC 10 100
NC 10 100
contrast
NC 10 100
NC 10 100
α-syn
NC 10 100
NC 10 100
contrast
NC 10 100
NC 10 100

Figure 7

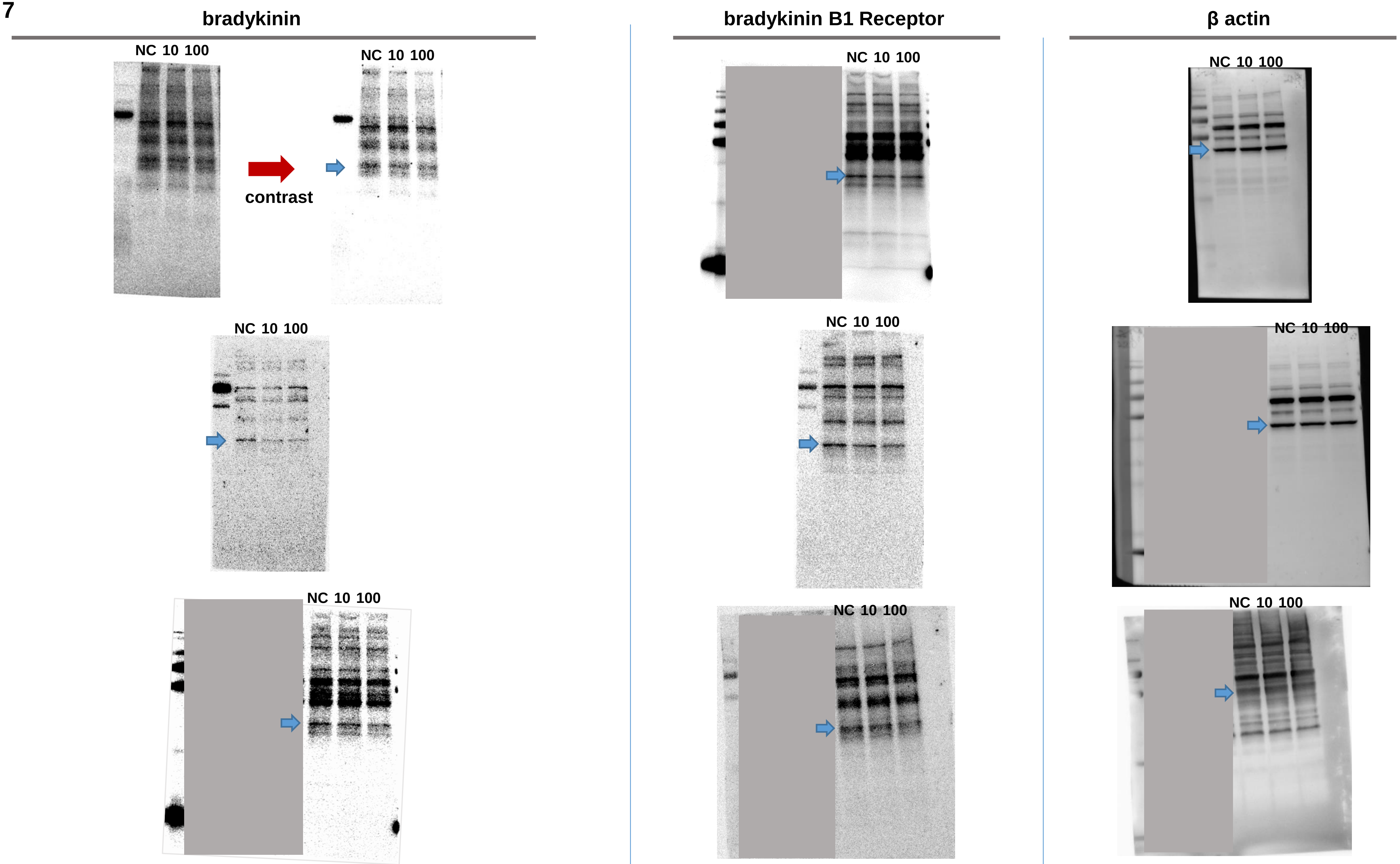
bradykinin
bradykinin B1 Receptor
β actin
NC 10 100
NC 10 100
contrast
NC 10 100
NC 10 100
NC 10 100
NC 10 100
NC 10 100
NC 10 100
NC 10 100
NC 10 100
